# Novel H^+^-Ion Sensor Based on a Gated Lateral BJT Pair

**DOI:** 10.3390/s16010014

**Published:** 2015-12-23

**Authors:** Heng Yuan, Jixing Zhang, Chuangui Cao, Gangyuan Zhang, Shaoda Zhang

**Affiliations:** 1 Science and Technology on Inertial Laboratory, Beihang University, No. 37 Xueyuan Road, Beijing 100191, China; zhangjixing@buaa.edu.cn (J.Z.); caochuangui@buaa.edu.cn (C.C.); zhanggangyuan@buaa.edu.cn (G.Z.); 2 Pen-Tung Sah Institute of Micro-Nano Science and Technology, Xiamen University, No. 422 South Siming Road, Xiamen 361005, China

**Keywords:** gated lateral BJT, MOSFET–BJT hybrid, ion sensor, ISFET

## Abstract

An H^+^-ion sensor based on a gated lateral bipolar junction transistor (BJT) pair that can operate without the classical reference electrode is proposed. The device is a special type of ion-sensitive field-effect transistor (ISFET). Classical ISFETs have the advantage of miniaturization, but  they are difficult to fabricate by a single fabrication process because of the bulky and brittle reference electrode materials. Moreover, the reference electrodes need to be separated from the sensor device in some cases. The proposed device is composed of two gated lateral BJT components, one of which had a silicide layer while the other was without the layer. The two components were operated under the metal-oxide semiconductor field-effect transistor (MOSFET)-BJT hybrid mode, which can be controlled by emitter voltage and base current. Buffer solutions with different pH values were used as the sensing targets to verify the characteristics of the proposed device. Owing to their different sensitivities, both components could simultaneously detect the H^+^-ion concentration and function as a reference to each other. Per the experimental results, the sensitivity of the proposed device was found to be approximately 0.175 μA/pH. This experiment demonstrates enormous potential to lower the cost of the ISFET-based sensor technology.

## 1. Introduction

The reference electrode that supplies the reference value for sensor data analysis is one of the most important components in a classical ion-sensitive transistor (IST) system. The IST has much been used in not only environmental detection, but also in biosensors [1,2,3,4]. With the development of sensor technology, semiconductor-based sensors have been extensively studied because of their perceived advantages such as low cost, small size, and ease of mass production. However, although the ion-sensitive field-effect transistor (ISFET) is based on the semiconductor-based sensor technology, the above advantages are not imputable to the current reference electrode technology. The primary reason is that the classical ISFET, which based on the metal-oxide semiconductor field-effect transistor (MOSFET) structure is difficult to operate under room temperature without gate bias, and the ISFET requires the reference electrode to supply a stable bias to the gate with a long lifetime, which requires Pt and Ag/AgCl reference electrodes [5]. These classical reference electrodes are additional components that increase the size of the device and make it inconvenient to use. Many researchers have improved the reference electrode by focusing on fabrication of the surface of the device [6,7], which is a well-known method for ISFET-based sensor fabrication, but this type of reference electrode is difficult to fabricate by the standard complementary metal-oxide semiconductor (CMOS) mass production process due to the bulky and brittle nature of the reference electrode materials. Moreover, the other fabrication processes cause a sharp increase in the production cost. In recent years, several new devices for ion detection without the reference electrode have been suggested owing to developments in organic materials and nano-technology [8,9]. They have successfully realized ion sensors without reference electrodes, but their cost and mass production fabrication issues have not been resolved yet.

The lateral BJT structure was first proposed in 1964 by Lin using a BJT process [10]. This special device combines a MOSFET and a BJT, and was developed for various power device applications [11]. In previous works, we developed a gated lateral BJT using the standard CMOS process for several sensor applications to achieve specific characteristics such as large dynamic range, high transconductance, and low gate bias [12,13,14,15,16,17].

In the CMOS process, the silicide treatment process has been widely used to form electrical contacts between the semiconductor material and the supporting interconnect structure [18,19,20]. In recent works, it was found that the silicide layer affects the characteristics of the gated lateral BJT. In this paper, the impact of the silicide layer on the gated lateral BJT was discussed. Based on this impact, we propose a H^+^-ion sensor that can be operated without the classical reference electrode. We fabricated a gated lateral BJT pair, which contains one gated lateral BJT with a silicide layer and another without the silicide layer. After comparing these two components, pH value detection experiments were performed and are discussed in this paper. According to the results, the proposed sensor can be operated without the reference electrode and the sensitivity was found to be approximately 0.175 μA/pH. The success of the proposed sensor has important implications for the development of advanced IST-based pH sensors and biosensors.

## 2. Experimental Setup

### 2.1. Fabrication of the Gated Lateral BJT Pair

**Figure 1:**
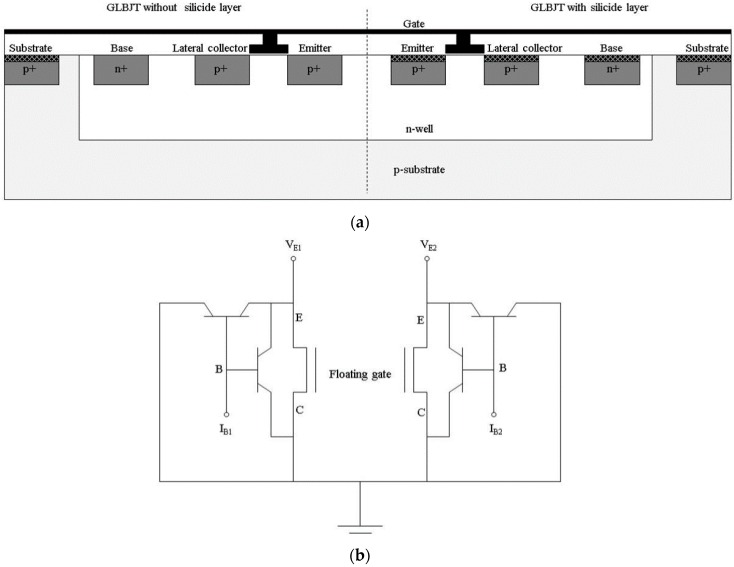
(**a**) Schematic and (**b**) equivalent circuits of the gated lateral BJT components with/without the silicide layer. The dotted line in (**a**) differentiates between the gated lateral BJT components with/without silicide layer.

The proposed sensor consists of one p-type gated lateral BJT with a silicide layer and one p-type gated lateral BJT without the silicide layer, both of which are of the same size and fabricated using a standard 0.35 μm CMOS process, as shown in Figure 1a. In this figure, GLBJT refers to the gated lateral BJT. A floating gate was fabricated on the top of the two components and is shared by the two components. A Si_3_N_4_ layer was fabricated on the top of the device for H^+^-ion concentration detection. The equivalent circuit of the proposed device is shown in Figure 1b. In this figure, the terms V_E1_, I_B1_, V_E2_, and I_B2_ denote the emitter (source) bias voltage and the base current of the two gated lateral BJT components, respectively. The symbols E, B, and C stand for the emitter (source), base, and lateral collector (drain) of the gated lateral BJT structures, respectively. The gated lateral BJT components with/without the silicide layer also share the n-well and a p-substrate. This type of structure can reduce the terminal volume and the errors, induced by the different base supply. In each component, there is one lateral p-n-p structure and one p-type MOSFET structure that can be operated. Besides, a vertical p-n-p BJT can be formed, but cannot be operate because the lateral collectors (drains) and the substrate were grounded. Each gated lateral BJT component could be operated under the MOSFET operation mode, BJT operation mode, and the MOSFET-BJT hybrid operation mode, which have been described in detail in previous works [12,13,14,15,16,17]. In other words, the MOSFET functional part is switched on by the gate bias supply, and the BJT functional part is switched on by the base current input. If the MOSFET and the BJT functional parts are switched on simultaneously, the gated lateral BJT is operated under the MOSFET-BJT hybrid operation mode. Joarder has proved that, in this mode, the emitter (source) current consists of a proportional MOSFET channel current and a bipolar current [21].

The fabricated device was embedded into a printed circuit board (PCB). All terminals were connected to the pins of the PCB.

### 2.2. pH Value Detection System Setup

**Figure 2:**
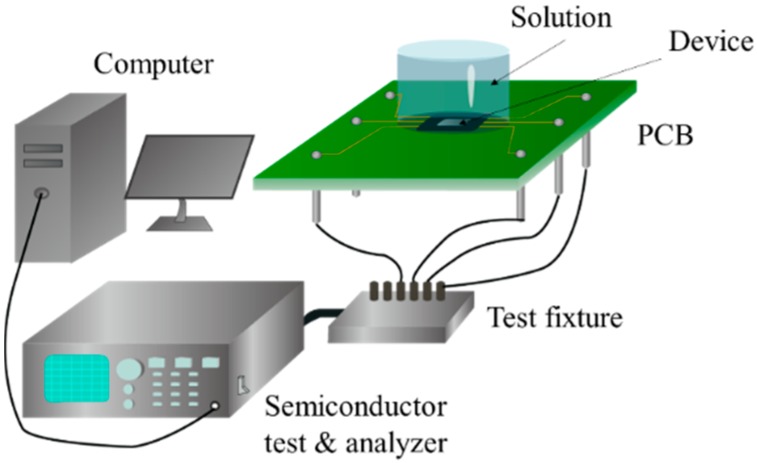
Schematic diagram of the experimental setup.

The schematic diagram of the pH value detection system setup is shown in Figure 2. It consists of the proposed sensor with PCB connections, a test fixture, a semiconductor test analyzer, and a control system implemented in a computer. The semiconductor test analyzer was used for supplying and detecting the input/output signals. The test fixture was not only used for connecting the semiconductor test analyzer to the sensor, but also to decrease the detection noise.

### 2.3. Comparison of the Electrical Characteristics of the Two Components with/without the Silicide Layer

According to the transconductance value, the gated lateral BJT has sensing capability [15], so the transconductance of the two components was verified. The gate bias and the base current were varied from –3 V to 5 V and from –50 μA to 20 μA, respectively. The emitter (source) bias supplied was 1 V. The lateral collectors (drains) and the vertical collectors (substrates) were grounded. The two gated lateral BJT components were then operated under the MOSFET–BJT hybrid mode, MOSFET mode and the BJT mode.

### 2.4. pH Value Detection

pH buffer solutions (with pH values of 4.00, 5.00, 7.00, 9.18, and 10.01) were used as the sensing target. For the setup to work, the two gated lateral BJT components functioned as references for each other, the bases and the floating gate were connected because of the specific structure, and both the emitters (sources) and the collectors (drains) were connected. The base currents were kept constant (–50 μA), and an emitter (source) bias was supplied with 1 V.

The sensing process can be divided into two steps: the first step follows the mechanism of the site-binding model theory that is based on the Si_3_N_4_ membrane [22,23,24,25,26]. Therefore, when the concentration of H^+^-ions varies, the potential of the floating gate is altered. This change affects the sensing process in the MOSFET channel. The second step is the sensing of the H^+^-ion concentration. The distinct advantage of this step is that the gated lateral BJT can operate below room temperature without the gate bias [16]. Therefore, based on the structural difference between the gated lateral BJT with the silicide layer and the gated lateral BJT without the silicide layer, the sensing properties are different. Finally, the sensing results are obtained by analyzing the data.

## 3. Results and Discussion

### 3.1. Comparison of the V_G_-I_E_ Curve of the Gated Lateral BJTs with/without the Silicide Layer

**Figure 3:**
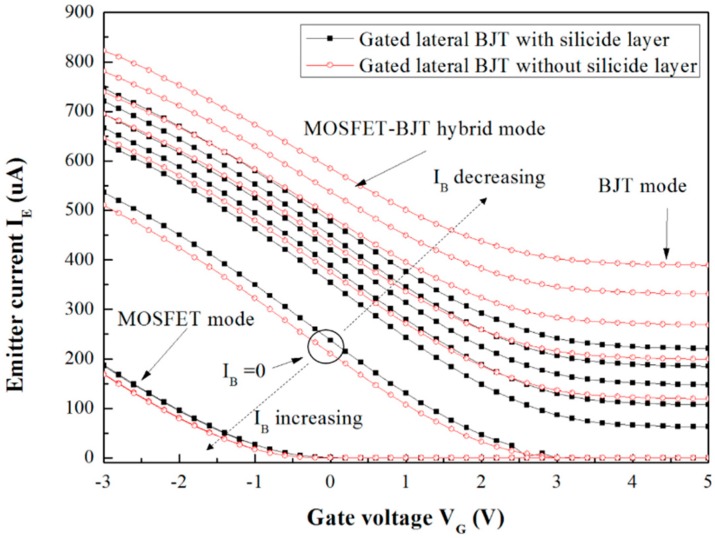
V_G_-I_E_ curve of the gated lateral BJT with/without silicide layer.

The transconductance curves of the gated lateral BJTs with/without the silicide layer were obtained from the V_G_-I_E_ curves of the two components, as shown in Figure 3. The classical operation modes of the two components are illustrated in this figure. The lower left curves refer to the MOSFET operation mode. The gate bias and the base current can affect the MOSFET functional parts and the BJT function parts were shut down or switched on, respectively. The portion on the right of the curves, in which the emitter currents have almost 0 μA output, indicate that the gated lateral BJTs were shut down.

In the case of the MOSFET functional parts, owing to the fact the gate bias primarily affects the channel current of the p-type MOSFET, the gate bias increases and the MOSFET channel current is gradually switched off, as shown in Figure 3. Moreover, in the case of the BJT functional parts, after the gate bias is increased, the percentage of the BJT current in the emitter current is increased too. In addition, according to the fact the transconductance of the BJT is higher than that of the MOSFET, the BJT effect and the emitter (source) current increase to produce higher V_G_-I_E_ curves as the base currents decrease. That is because the BJT has higher transconductance characteristics than the MOSFET. Furthermore, in the MOSFET operation mode, the curves did not change according to the base current because the BJT functional parts were switched off.

Following the results, the gated lateral BJT component without the silicide layer exhibited lower threshold voltage in the MOSFET operation mode and higher emitter (source) current during the operation of the BJT functional parts. This is because, first, the silicide layer reduces the doping depth, which reduces the BJT effect. Besides, the MOSFET functional part in the gated lateral BJT component without the silicide layer is nearer to the gate than that in the gated lateral BJT component with the silicide layer. Second, the percentage of the BJT current in the gated lateral BJT component without the silicide layer shows more dominance than the gated lateral BJT component with the silicide layer.

**Figure 4:**
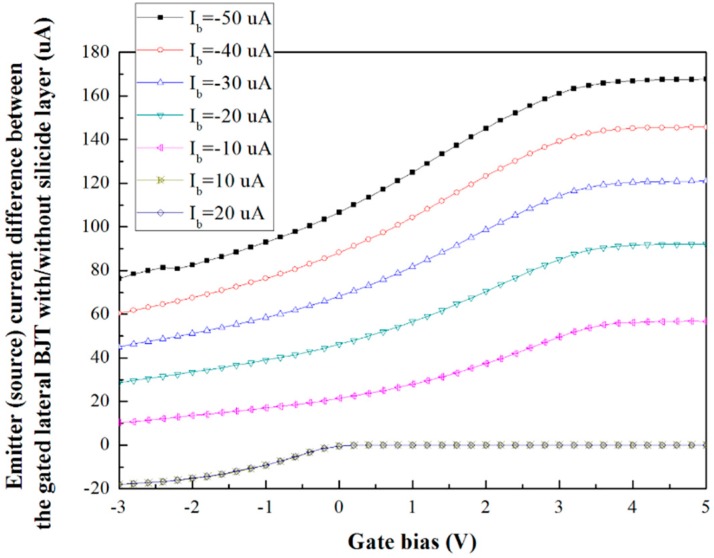
V_G_-I_E_ curve difference between the gated lateral BJT components with/without silicide layer.

The V_G_-I_E_ curve difference between the gated lateral BJTs with/without the silicide layer can be illustrated as shown in Figure 4.

In accordance with these results, the difference between the two components increased as the base current decreased. A base current of −50 μA was used in the following ion detection experiment.

### 3.2. The V_G_-g_m_ Curve Properties of the Two Sensor Components

**Figure 5:**
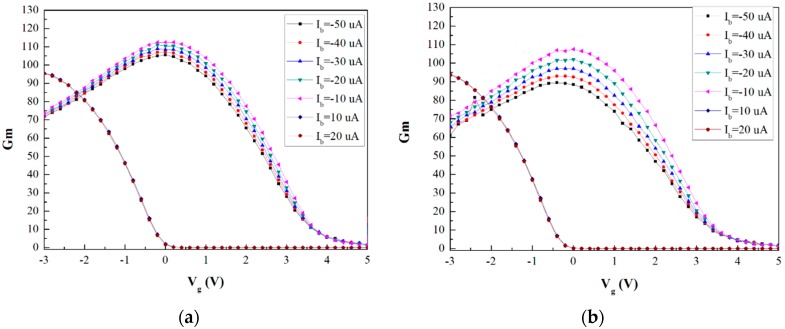
V_G_‒*g_m_* curve of the gated lateral BJT components with (**a**) and without (**b**) silicide layer.

According to the V_G_-I_E_ curve and Equation (1), the transconductance curve of the proposed device can be obtained, as shown in Figure 5:

(1) $$g_{m}=\partial I_{e}/\partial V_{GC}$$

where *g_m_* is the transconductance, *∂_IE_* and *∂V_GC_* are the changes of the emitter (source) current and the gate voltage, respectively.

According to Figure 5, the gated lateral BJT with the silicide layer had a higher transconductance value than the one without the silicide layer at constant base current. However, its transconductance difference was smaller than the one without the silicide layer under varying base current conditions. This is because the BJT effect was dominant when the gated lateral BJT lacked the silicide layer, as described in Section 3.1. Since the sensitivity of the two components was primarily decided by the MOSFET functional parts, the gated lateral BJT component without the silicide layer has lower sensitivity. Moreover, in this case, the percentage of carriers in the BJT and MOSFET functional parts could be controlled easily. It was due to this phenomenon that the transconductance difference of the gated lateral BJT component without the silicide layer was smaller than that with the silicide layer.

The sensitivity of a sensor can be calculated as Equation (2):

(2) $$\text{S}=\partial I_{output}/\partial V_{input}$$

According to the experimental setup, the input signal was the gate bias, which changed with the H^+^-ion concentration of the target solution; the output signal was the emitter (source) current. Therefore, the transconductance behavior can reflect the sensitivity of the sensor. Subsequently, the sensitivity of the gated lateral BJT components with/without silicide layer is difference on the basis of Figure 5.

### 3.3. pH Value Detection Based on Proposed Device

**Figure 6:**
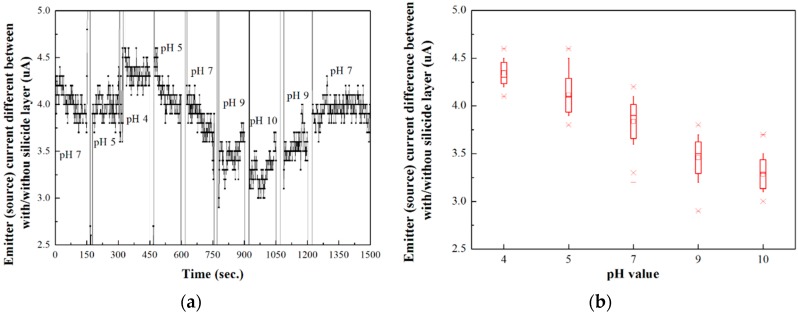
Plot of the H^+^-ion detection using (**a**) the proposed gated lateral BJT pair device against time and (**b**) against pH value.

pH value detection experiments were carried out using the variation of the pH value of the target solution with time. The difference between the two gated lateral BJT components was defined as the sensitivity of the sensor. The experiments were performed from a pH value of 7.00 down to the pH value of 4.00, and then, the pH was increased up to a pH value of 10.01, and then back to the pH value of 7.00, as shown in Figure 6a. The results between two pH values were obtained on the device as and when there was a change in the concentration of H^+^-ions. The reversibility of the proposed device was also supported.

In order to analyze the results, Figure 6b was extracted from Figure 6a. Figure 6b shows the resultant emitter (source) current difference curve against the pH value without the reference electrode. According to the results, the proposed device can be used for H^+^-ion detection without the reference electrode. The sensitivity was calculated as approximately 0.175 μA/pH. As the pH value increased, the concentration of the H^+^-ion in the buffer solutions decreased. This induced a negative potential in the floating gate. According to Figure 4, the emitter (source) current difference between the two gated lateral BJT components decreased.

## 4. Conclusions

A gated lateral BJT pair that consists of two gated lateral BJT components, one with a silicide layer and the other without the silicide layer, was demonstrated for H^+^-ion detection. The proposed sensor can be operated without a reference electrode. In order to prove the possibilities of this approach, the V_G_-I_E_ and the transconductance of the gated lateral BJT components with/without the silicide layer were compared. The results show that the BJT effect of the gated lateral BJT without the silicide layer was more distinct than that of the one with the silicide layer. In addition, the gated lateral BJT with the silicide layer had a higher transconductance value and a smaller transconductance difference with varying base currents. After the phenomenon was discussed, an ion detection experiment was performed using a pH buffer solution and a Si_3_N_4_ sensing membrane. The two gated lateral BJT components function as references for each other. The experiments proved that the proposed device could successfully detect H^+^-ions. This device this achieved the detection of H^+^-ions without the reference electrode and was fabricated by the standard CMOS process. It demonstrated a significant cost reduction for the ISFET-based sensor technology. Furthermore, this device can be used as a prototype in similar research fields. In the future, the structure of the device will be optimized and apply to the biosensors.
